# Mid-Pregnancy Ultrasonographic Cervical Length Measurement (A Predictor of Mode and Timing of Delivery): An Observational Study 

**Published:** 2018-03

**Authors:** Jabacsmick Sharmila Thangaraj, Syed Habeebullah, Sunil Kumar Samal, Sunita S Amal

**Affiliations:** Department of Obstetrics and Gynecology, Mahatma Gandhi Medical College and Research Institute, Pondicherry, India

**Keywords:** Cervical Length, Mode of Delivery, Timing of Delivery, Prediction

## Abstract

**Objective:** Even though cervical length is considered as predictor of timing and mode of delivery, it is not used as a screening tool in low risk asymptomatic population. This study was carried out with the intention to know the timing and mode of delivery in asymptomatic low risk women using second trimester ultrasonographic cervical length measurement and predict the risk of pretermlabor, prolonged pregnancy and need for caesarean section. 1) To determine the association between cervical length at mid-pregnancy and timing of delivery. 2) To determine the usefulness of mid-pregnancy ultrasonographic cervical length measurement in predicting mode of delivery.

**Materials and methods:** Transvaginal sonography was performed to measure the cervical length between 20-24 weeks of gestation. These patients were followed till delivery to assess the gestational age at delivery and mode of delivery.

**Results:** Totally 237 patients were recruited of which 173 satisfied the inclusion criteria. Out of 15 patients with cervical length less than 3cm, 14(93.33%) had preterm delivery. Postdated pregnancy was observed in 45(90%) out of 50 patients with cervical length more than 4cm. In the group with cervical length less than 3cm, 12 (80%) delivered vaginally. Among cervical length more than 4cm group 24 (48%) required cesarean section.

**Conclusion:** Cervical length of less than 3cm measured between 20-24 weeks of gestation is associated with preterm births and favours vaginal birth whereas, cervical length of more than 4cm is associated with postdated pregnancy and increased incidence of cesarean section.

## Introduction

Preterm birth is a common obstetric problem accounting for 11.4% of deliveries in 2011 and prolonged pregnancy ranged from 4-14% (1). Cesarean section (C.S) rate was 15.4% in 2014- 15. Preterm birth remains a major cause of neonatal morbidity and mortality due to complications like necrotizing enter colitis, intraventricular hemorrhage, respiratory distress syndrome and neurological deficit (1). There are various methods to predict preterm labor like 1) cervical length 2) fetal fibronectin 3) cortisol level 4) placental hormone level and 5) non invasive electromyography (2- 6).

Post dated pregnancy has its own complications like fetal macrosomia, oligohydramnios, increased risk of meconium stained liquor and operative intervention (1).

Predictive measures of postdated pregnancy include cervical length measurement by ultrasonography and measurement of fetal fibronectin, cytokine and nitric oxide concentration in cervicovaginal secretions (7). Some studies have shown that cervical length assessed by transvaginal ultrasonography could predict the possibility of prolonged pregnancy in nulliparous women (7, 8). It has also been noted that there is an association between cervical length during mid pregnancy and cesarean section due to non progress of labor at term. 

Even though cervical length is considered as predictor of timing and mode of delivery, it is not used as a screening tool in low risk asymptomatic population. The aim of this study is to predict the timing and mode of delivery using mid-pregnancy ultrasonographic measurement of cervical length.

## Materials and methods

The study was carried out in the Department of Obstetrics and Gynecology, Mahatma Gandhi Medical college and Research Institute, Pondicherry (INDIA). The recruitment for the study was done from December 2015 to April 2017. All primigravidae attending antenatal clinic with singleton pregnancy with no comorbidities at 20-24 weeks of gestation were included in the study.

The exclusion criteria were fetal abnormalities, associated medical complications like hypertension, diabetes, short stature, elderly primigravidae, teenage pregnancy, early trimester bleeding, conception afterassisted reproductive techniques (IUI, IVF) and induction of labor before 40 weeks of gestation.

Detailed history was obtained from the patients at recruitment and they were excluded as per exclusion criteria. After obtaining consent, they were subjected to transvaginal ultrasonography between 20-24 weeks of gestation. Cervical length was measured by one of the two authors. These measured cervical lengths were kept confidential from the obstetrician conducting delivery. Subsequently, those who developed complications like GDM, GHTN, malpresentation or who required induction of labor before 40 weeks for other complications like IUGR, oligohydramnios, decreased fetal movements were excluded from the study. These patients were given routine antenatal care according to hospital protocol.

The outcome of each pregnancy e.g.; whether preterm, term up to 40 weeks or post dated (after 40 weeks till 41 + 6 weeks), whether labor was spontaneous or induced, mode of delivery whether vaginal or cesarean section and indication for C.S were recorded. These results were correlated with mid-trimester ultrasonographic cervical length measurements.

For the purpose of our study, cervical lengths were categorized into four groups: 1) cervical length ≤ 2cm, 2) cervical length between 2.1-3 cm , 3) Cervical length between 3.1-4 cm and 4) Cervical length more than 4 cm. Preterm labor was defined as the onset of labor after 28 weeks and before 37 completed weeks. Term pregnancy was defined as gestational age between 37 and 42 completed weeks. Postdated pregnancy was the pregnancy that lasted more than 40 weeks of gestation (9). Institutional ethical committee approval was obtained. The minimum sample size was calculated to be 140.

***Statistical analysis:*** Data like cervical length, timing of delivery, onset of labor, mode of delivery were analysed by chi-square test and p value less than 0.05 was considered to indicate statistical significance. Indications for C.S. were analysed using percentage. The statistical software namely SPSS 17.0 was used for analysis of the data. Microsoft word and excel have been used to generate tables etc.

## Results

Two hundred and thirty seven antenatal patients were recruited from December 2015 to April 2017 formed the subjects of the study. Out of these 237 patients, 64 were subsequently excluded as they developed complications like gestational hypertension and gestational diabetes mellitus that required induction of labor before 40 weeks of gestation for complications like oligohydramnios, fetal growth restriction, PROM etc. Finally, 173 patients were included in the study and their measured cervical lengths were correlated with their timing and mode of delivery. Mean gestational age at which cervical length was measured was 22 weeks and 6 days.

While comparing demographic characters, majority of the patients (66.47%) were between 20-25 years of age. Over 74% of the study population belonged to middle class. Most of the patients had cervical length between 3.1- 4cm (62.4%). There was no patient with cervical length less than 2 cm (Table 1).

**Table 1:** Second trimester cervical length

**Cervical length**	**No of patients**	**Percentage**
≤ 2cm	0	0
2.1-3 cm	15	8.7
3.1-4 cm	108	62.4
> 4cm	50	28.9
TOTAL	173	100

Table 2 shows the association between cervical length and timing of delivery. Mean Gestational ages at which patients delivered were 35 + 4, 39, 40 + 3 weeks when their cervical lengths were 2.1-3, 3.1-4 and > 4cm respectively. The correlation between gestational age and mode of delivery was shown in Table 3. In patients with cervical length 2.1 -3 cm, 80% delivered vaginally where as in those with > 4 cm cervical length 48% had undergone cesarean section.

**Table 2:** Association between cervical length and timing of delivery

**Cervical length**	**Total**	**Preterm delivery**
	**(No.)**	**NO (%)**
≤ 2cm	0	0
2.1-3cm	15	14(93.3%)
3.1-4cm	108	8(7.4%)
> 4cm	50	0

In Table 3, details regarding number of patients induced and their mode of delivery are tabulated. While analysing the indications for cesarean section in cervical length more than 4 cm group, it was observed that 10 (41.67%) were performed for arrest of dilatation.

## Discussion

This study shows significant association between the cervical length during the mid-pregnancy period and timing of delivery. Fourteen out of 15 with cervical length less than 3cm delivered preterm with mean gestational age at delivery being 35 weeks and 4 days (± 7 days). This association becomes stronger as the cervical length increased; 90% of patients with cervical length more than 4 cm delivered postdates after 40 weeks and before 42 weeks of gestation with mean gestational age at delivery being 40 weeks and 3 days (± 3 Days). Tanvir et al found that 81.25% of patients with short cervix delivered spontaneously as preterm and concluded that transvaginal sonography is a sensitive method which was simple and cost effective in predicting the risk of preterm delivery (8). In another study in 2015 it was reported that among patients with short cervix, the risk of preterm labor was 66.7%.^9^Boelig et al, used cut off of 3.7cm for cervical length and found that there was a two fold increased risk of prolonged pregnancy in those with cervical length more than 3.7cm when compared with those with cervical length less than 3.7cm (10).

In the present study, 80% of patients with length of the cervix between 2.1 and 3cm delivered vaginally. Among cervical length more than 4cm, percentage of women delivered vaginally was 52% and abdominally was 48%. This was higher when compared to the overall C.S rate across all cervical lengths (38.15%).In the group with > 4 cm cervical length, 50% required induction of labor and arrest of dilatation was a common indication (41.67%) for C.S in them whereas in 3.1-4cm group it was 17.95%.No CS was done for arrest of dilatation in cervical length less than 3cm group. These findings agree with the findings of kalu et al who found that long cervical length at mid- pregnancy can predict the possibility of cesarean delivery and concluded that the cervical length in mid pregnancy can be of value in predicting the mode of delivery (9).

## Conclusion

Based on the present data, we conclude that: 1) Short cervix between 2.1-3cm measured at 20 to 24 weeks of gestation is significantly associated with preterm delivery and long cervix of more than 4cm is significantly associated with postdated delivery. 2) Mid pregnancy cervical length measurement is useful in predicting the possibility of vaginal delivery in women with cervical length between 2.1 and 3cm and risk of cesarean section in women with long cervix of more than 4 cm.

**Table 3:** Cervical length and mode of delivery

**Cervical length**	**Total**	**SVD**	**Instrumental delivery**	**LSCS**	**P value**
	**NO**	**NO. (%)**	**NO. (%)**	**NO. (%)**	
≤ 2cm	0	0	0	0	0.227
2.1-3cm	15	12(80%)	0	3(20%)	
3.1-4cm	108	65(60.1%)	4(3.7%)	39(36.2%)	
> 4cm	50	24(48%)	2(4%)	24(48%)	

## Conflict of Interests

Authors have no conflict of interests.
